# Radiobiology of the C3H Mouse Mammary Carcinoma: Effect of Immunogenetic Factors on the Radiosensitivity of the Tumour Treated In Situ

**DOI:** 10.1038/bjc.1954.31

**Published:** 1954-06

**Authors:** A. Cohen, L. Cohen


### RADIOBIOLOGY OF THE C3H MOUSE MAMMARY CARCINOMA: EFFECT OF IMMUNOGENETIC FACTORS ON THE RADIO-SENSITIVITY OF THE TUMOUR TREATED *IN SITU*.

A. COHEN AND L. COHEN.

*From the Experimental Oncology Laboratory, Radiation Therapy Department, Johannesburg, General Hospital.*

Received for publication April 20, 1954.

IT has been shown that the curative dose in irradiation of tumours is a relative quantity dependent upon the state of resistance of the host. Having established the median effective dose for the treatment of the C3H mammary adenocarcinoma *in situ*, it was subsequently demonstrated that when the resistance of the tumour-bearing animals was deranged by total-body irradiation, a significant decrease in the radiosensitivity of the tumour occurred (Cohen and Cohen, 1953*a*, 1953*b*). Conversely, a state of relative genetic resistance in the host can result in a marked increase in the radiosensitivity of a tumour. Oughterson, Tennant and Lawrence (1940) showed that $F_2$ hybrids, a cross between low-tumour and high-tumour mouse strains, bearing a mammary carcinoma originating in the high-tumour parent stock, exhibited a natural resistance evidenced by a moderate incidence of of spontaneous regressions, and were readily cured with a relatively small dose of radiation known to be almost ineffective against the same tumour in the susceptible parent strain.

In the following experiments, the curative dose for the C3H mammary carcinoma growing in reciprocal $F_1$ hybrids derived from high mammary tumour C3H strain and low-tumour strain mice is determined. All individuals of the $F_1$ generation are susceptible to transplants of the parent strain tumour and show no spontaneous regressions, yet the relative genetic incompatibility of this host tumour relationship should, if radiosensitivity is dependent on host-resistance result in a significantly lower curative dose than that required by the same tumour in the parent strain. Any similar effects attributable to the milk-factor could also be detected by comparison between the reciprocal hybrid groups.

### MATERIALS AND METHODS.

### *Animals.*

In a preliminary experiment, 80 reciprocal $F_1$ hybrids derived from our high mammary tumour strain C3H/Cg and a low-tumour heterogeneous albino stock, were used. Since the results of treatment in these hybrid mice were so variable as to obscure statistical comparison between the reciprocal groups, all subsequent experiments were done with reciprocal $F_1$ hybrids derived from the homogeneous low-tumour strain CBA/No (supplied by Dr. D. J. Nolte, Department of Zoology, University of the Witwatersrand).

For the sake of brevity, the hybrid (CBA $\times$ C3H)$F_1$, considered to be factorfree, will, in this paper, be designated as FFC, and the reciprocal factor-harbouring (C3H × CBA)$F_1$ as FHC.

All parent strain and hybrid mice were housed in the same environment and maintained on a standard laboratory chow diet with tap water *ad libitum*. It was found expedient, in view of the temperamental breeding habits of both CBA and C3H females, to isolate routinely all pregnant mice until after the weaning of the litters. Consequently, no $F_1$ hybrid was ever in contact with the father, which, as has recently been shown by Peacock (1953), reduces the probability of " infection " in the FFC with milk-factor from the C3H male parent.

The incidence of spontaneous mammary tumours in old factor-harbouring (C3H × stock albino) $F_1$ females, which were observed for over a year and inadvertently force-bred, was at least 70 per cent. No mammary tumours were noted in the corresponding, similarly treated, factor-free females, and it was considered that the presence of the milk-factor in the C3H male parent exerted little or no influence on this group. The reciprocal hybrids of the CBA–C3H cross were observed for considerably shorter periods, during which some spontaneous tumours had already made their appearance in FHC females aged 7 to 9 months, while none had appeared in the FFC reciprocals.

*Tumour.*

The tumour tissue used in the following experiments consisted of established homoplasts of the C3H mammary adenocarcinoma which had not been passaged more than once or twice from spontaneous tumours arising in females of the C3H/Cg strain. In order to avoid undue variation as a result of prolonged residence in hybrid hosts, the tumour was transplanted directly from the C3H to the $F_1$ hybrids except, in one experiment, where a tumour growing in a hybrid was deliberately used. In each series of tumour inoculations, collateral groups of C3H mice were implanted as controls. Recipients consisted of random groups of both sexes, weighing approximately 20 to 25 g. The technique of homoiotransplantation has been described in detail in the first paper of this series (Cohen and Cohen, 1953*a*). Tumour " takes " were 100 per cent in all $F_1$ hybrids, with no spontaneous regressions.

*Radiation procedure.*

Technical factors and techniques of treatment of the tumour *in situ* used in these experiments are identical with those reported in the previous paper (Cohen and Cohen, 1953*b*). The tumour, retracted from the body onto a wax backing, is fitted into the mouth of a 2 cm. diameter open-end applicator, FSD = 25 cm., and treated at 240 kV. with no added filters, HVL = 0·34 mm. Cu., at a dose-rate of 500 r/min. in air.

*Tumour size and growth rate.*

The rate of growth of the implanted tumour before treatment differed sharply in the various genetic groups. Assuming, for the purpose of this comparison, an exponential increase ($V.e^{gt}$) of tumour volume ($V$) with time in days ($t$), the growth constant ($g$) was estimated for each mouse from measurements of tumour volume at the time of treatment, and then averaged for all animals in each genetic category. In the case of the FHC mice, the growth constants corresponded to an average value of $g = 0{\cdot}18$ ($\pm$ 0·015), which is not significantly different from that of the C3H parent, in which $g = 0{\cdot}20$ ($\pm$0·014) day$^{-1}$. In the FFC group, however, $g = 0{\cdot}13$ ($\pm$ ·015), which differs significantly (p = 0·01) from the two other groups. In the factor-free hybrid (FFC), therefore, the growth rate is significantly slower than that for the same tumour in factor-harbouring mice, whether homozygous (C3H) or hybrid (FHC). This observation corroborates Barrett and Morgan's (1949) findings that a maternal influence significantly accelerates the growth rate of the C3H tumour in $F_1$ hybrid mice.

The period elapsing between the appearance of a palpable " take " and the time at which the tumour was large enough for treatment by the techniques described, necessarily ranged from 10 to 60 days, and was, in general, longer in the case of FFC than among FHC mice. Further, as a result of the difference in growth rates in the two genetic subgroups, it was not always possible in this experiment to maintain a strictly constant tumour size at the time of irradiation. While the possibility that the size factor might affect the radiosensitivity cannot be entirely discounted, previous observations on the C3H host had shown that, provided the tumour was uniformly irradiated, the radiosensitivity was apparently independent of tumour size within the limits imposed by size of the treated portal.

In no treated tumour did the greatest diameter exceed 20 mm., nor the maximum thickness exceed 10 mm. Correcting for the fact that the bulk of the tumour was allowed to bulge into the open end of the applicator, it can be shown from published depth-dose tables for this quality of radiation (Braestrup, 1944) that the tumour doses range from a maximum of 111 per cent of the given air-dose at the upper pole, to an absolute minimum of 89 per cent at the base of the largest tumours treated. This difference adds little to the inherent dosimetric errors, and is insignificant in comparison to the large differences in radiosensitivity induced by the experimental variables.

All tumours were roughly spheroidal in shape, and their volumes ($V$) could be estimated by measurements *in situ* in the anaesthetised mice at the time of treatment, from the formula $V = \frac{\pi}{6} ab^2$, where $a$ is the axial length and $b$ the equatorial diameter of the spheroid. Inevitably, the average tumour-size at the time of treatment was somewhat larger in the FHC than in the FFC group, while the exceedingly skew distribution of the volumes within each group renders quantitative comparison difficult. The histograms in Fig. 1 show the reasonably symmetrical distribution in both genetic subgroups when a logarithmic transformation of the volume scale is employed. The mean-log of the volume in cubic millimeters of the FHC group is 2·12 ($\pm$ 0·08), while the mean-log-volume for the FFC group is 1·85 ($\pm$ 0·08), which is significantly smaller.

While unequivocal comparisons between the C3H and hybrid groups could be made with confidence, it was considered possible that, as a result of the variations in growth rate within the hybrid hosts, smaller tumours might be more readily cured, and the FFC group consequently biased in favour of a higher cure-rate. A more objective test of the maternal influence *per se* could, however, be obtained by eliminating from the analysis sufficient animals from the opposite extremes of the distributions, in this case the 6 largest tumours in the FHC and the 6 smallest tumours in the FFC mice (shaded areas in Fig. 1), in order to equate the geometric means of the two groups, thus permitting comparison between tumours of matched sizes.

*Experimental design.*

Since the object of this investigation is to determine the effect on the radio-sensitivity of the parent strain tumour of genetic differences in the host, including possible maternal influences, it was necessary to estimate the magnitude and significance of differences in response of irradiated tumours in the parent strain and in the reciprocal hybrid groups respectively. Accordingly, groups of mice in the various genetic categories were treated at several dosage levels and the

FIG. 1.—Histogram showing distribution of tumour sizes at the time of treatment in reciprocal hybrids. Black areas : Cured tumours. White areas : Non-cures. Shaded areas : Mice eliminated from statistical analysis of matched mean log-volumes. M : Common geometric mean and its standard error.

proportion of cures in each group analysed by the probit method (Fig. 2), estimating for each category, the median effective dose and its standard error, the individual variance, and the linearity or otherwise of the probit regression line.

In the preliminary experiment with the heterogeneous hybrids, the excessive individual variation and obviously non-normal distribution obscures inter-group differences and renders detailed statistical analysis unprofitable, although a rough estimate of the $LD_{50}$ from pooled data can be obtained by inspection of Fig. 2. In the second experiment a total of 72 mice from both genetic subgroups (FHC and FFC) were irradiated at three dose levels. The proportion of cures in each group was tabulated (Table I) and plotted on the log-probit co-ordinates of Fig. 2, together with comparative data previously determined for the parent strain. This design is analogous to a six-point assay, in which both linearity and parallelism of the regression lines can be tested. The magnitude of the parameters and the significance of the inter-group differences were computed by Finney's (1952) methods.

A third experiment was designed to determine whether the increased radiosensitivity of the tumour in $F_1$ hybrids was an inherent change in the tumour cells *per se*, or a reflection of host-resistance. The possibility of a change in tumour

FIG. 2.—Probit diagram showing response of the C3H carcinoma growing in various situations. (A) Control series treated in homozygous C3H mice *in situ*. (D) Irradiated *in situ* in heterogeneous hybrid mice. (E) Irradiated in factor-harbouring (FHC) mice. (F) Irradiated in factor-free (FFC) mice. The increased radiosensitivity of the tumour associated with genetic diversification in the host is evident.

cells growing in a relatively alien environment was suggested by Barrett and Deringer's (1952) observation that a permanent adaptive change occurred in the C3H mammary carcinoma following one-sub-passage through susceptible $F_1$ hybrids. Since the object of this experiment was to demonstrate any inherent acquired radiosensitivity in the tumour, a small homoplast (first passage from a C3H mouse), growing slowly in an FFC host, which had attained a volume of only 30 $mm^3$. in 60 days after implantation, was excised and transplanted into mice of the three genetic categories, C3H, FHC and FFC. The donor was also re-implanted with this tumour. " Takes " were 100 per cent in all groups. When each tumour had reached a standard size, about 1 cm. in its greatest diameter, it was irradiated *in situ* with a dose of 4200 r. The re-implanted tumour in the FFC donor was also irradiated at this dose, and cured. Therefore, if the acquired radiosensitivity was a permanent adaptive change in the tumour cells capable of being carried over into the next transplant generation, it could be expected that the cure-rate of grafts originating from this tumour at the dose given would be about 75 per cent in each genetic group, or possibly even higher.

Conversely, if the resistance of the host was the deciding factor, the cure-rates in each group would be comparable with results of the preceding experiments.

### RESULTS.

*Preliminary experiment.*

The results of treatment *in situ* at 6 different dose levels of the C3H mammary tumour growing in reciprocal $F_1$ hybrids derived from the C3H strain and the mixed albino stock are shown in Fig. 2, Line D. The proportion of cures with respect to dosage is based on pooled data from 80 mice, approximately equally divided between both reciprocal crosses. It is immediately apparent that the results do not follow a linear probit, thus reflecting the heterogeneity of the material. Inspection of the graph suggests that a proportion of these mice show a marked response to comparatively low doses, the median effective dose being in the region of 4500 r, which is obviously lower than that previously reported for the same tumour in the inbred parent strain. Due to the heterogeneity of the data, no evidence for the existence of a possible maternal influence on the response of the tumour could be adduced.

*Second experiment.*

Table I demonstrates the response to treatment *in situ* at 3 dose levels of the FHC and FFC hybrids bearing the C3H carcinoma. It will be noted that a significant proportion of cures is obtained in both reciprocal groups with relatively low dosage (4200 r) known to be completely ineffective in the C3H parent strain, the factor-free group having consistently higher cure rates at all three dose levels.

These results are plotted in Fig. 2, and the probit regression lines derived for each reciprocal group are shown. The radiosensitivity of the tumour growing in the genetically heterozygous environment of the FFC host, excluding the complicating influence of maternal factors, is shown by Line F in Fig. 2. This line is virtually parallel to that previously obtained in the parent C3H strain (Line A on the diagram), the coefficient of variation being 10 per cent. The median effective dose ($LD_{50}$) is found to be 3950 ($\pm$ 110*)r, compared to 5700 r previously reported in the C3H. The relative radiosensitivity of the tumour in the C3H compared to FFC mice, virtually a measure of the effect of the changed genetic milieu, can be expressed as the ratio of the two $LD_{50}$'s, $\frac{5700}{3950} = 1{\cdot}44$ ($\pm$ 0·05*). The magnitude of this ratio compared to its standard error shows the difference in radiosensitivity of the two groups to be highly significant ($p < 0{\cdot}0001$).

TABLE I.—*Treatment of the C3H Adenocarcinoma Growing in Reciprocal $F_1$ Hybrids (C3H with CBA Strains).*

| | Factor-free hybrid (FFC). | | | Factor-harbouring hybrid (FHC). | | | |
|---|---|---|---|---|---|---|---|
| Dose (r). | Number of mice. | Number cured. | Cures (per cent). | Number of mice. | Number cured. | Cures (per cent). | C3H cures (per cent). |
| 5000 | 8 | 8 | 100 | 8 | 3 | 37 | 8 |
| 4200 | 17 | 13 | 76 | 17 | 5 | 29 | 0·1 |
| 3500 | 11 | 2 | 18 | 11 | 0 | 0 | 0·01 |
| Total | 36 | 23 | 64 | 36 | 8 | 22 | 0·2 |
| Total (matched volumes) | 30 | 17 | 57 | 30 | 8 | 27 | — |

The corresponding data for the FHC group is shown by Line E in Fig. 2. Not only is the cure-rate, in the presence of the milk factor, considerably less than that of the factor-free mice, though still greater than that of the C3H parent, but the usual sharp response to increasing dosage has become much less sensitive, as evidenced by the considerably flatter slope of the regression line. This change is not due to fortuitous fluctuations in cure-rates, as a $\chi^2$ test of the data reveals a highly significant departure from parallelism with the other groups. The median, effective dose for the tumour growing in FHC mice appears to be 5100 ($\pm$ 400*) r with the unusually large coefficient of variation of 25 per cent. It must be assumed, therefore, that the maternal influence is not uniform and stochastically independent of other factors. A similar result was noted in the treatment of radiation-attenuated homoplasts growing in the parent strain (Cohen and Cohen, 1954).

At the $LD_{50}$ level, the relative radiosensitivities, evidenced by the dosage ratio between the C3H and FHC groups is 1·13 ($\pm$ ·07*), and that between the FHC and FFC groups is 1·29 ($\pm$ ·08*). The former ratio, compared with its standard error, indicates that the genetic effect *per se*, in spite of the complicating maternal influence, is probably significant ($p = 0{\cdot}05$) ; while the latter shows that the effect attributable to the milk factor alone within the hybrid mice is highly significant ($p < 0{\cdot}001$). The relative importance of these ratios, however, necessarily varies with the curative level tested, the maternal influence having the greater effect at doses above the median effective range.

In order to eliminate any possible bias introduced by the greater average volume of the tumours in the FHC mice compared with those of the FFC group, a small number of animals bearing tumours of extreme size were discarded in the manner previously described, so as to equate the mean log-volumes of the two groups, and the probit analyses repeated on the remaining matched tumours. This correction, however, does not alter the magnitude of the parameters or affect materially the significance of the differences between them. Further, comparison of the proportion of tumours cured within each class-interval of Fig. 1, shows the large relative preponderance of cures in the FFC group. It will also be noted (Table I) that among mice with matched mean volumes, a total of 17 out of 30 FFC's, but only 8 of 30 FHC's, were cured. This difference is significant ($p = 0{\cdot}02$).

On the other hand it is also apparent from Fig. 1 that within each genetic subgroup, the cured tumours tend to a smaller volume than the non-cures. These differences, however, are not significant, since the experiment was not designed to test the effect of tumour volume *per se* on radiosensitivity and contains insufficient data for this purpose. The question of the relative importance of genetic factors and of tumour size *per se* in determining radiosensitivity, is probably meaningless, since the smaller, more slowly-growing homoplast may itself be a manifestation of genetically-conditioned host-resistance (Eichwald, 1953). From the foregoing it may be concluded that, although the presence of the maternal factor enhances tumour growth-rates (Barrett and Morgan, 1949), and larger tumours may be less readily cured than smaller ones, nevertheless the maternal influence does affect radiosensitivity of the C3H tumour growing in reciprocal hybrids, even when the effect of gross variation in size is eliminated. Both genetic and extra-chromoso-

* Standard errors of medians and of ratios of medians.

mal factors, therefore, materially affect the response of a tumour homoplast irradiated *in situ*.

Subsequent challenge with a second inoculum in those hybrids of both reciprocal groups, previously cured of the tumour, resulted in active growth in all cases. It was also interesting to note, in the course of the experiment, that the spontaneous mammary tumours in factor-harbouring $F_1$ females occurred regardless of whether the animal had previously been cured or not.

*Third experiment*

Table II shows the results of the third experiment, in which a " radiosensitive " homoplast, growing in an FFC and later cured with 4200 r, was sub-passaged through mice of the three genetic categories and treated with the same dose. The cure-rates of these tumours in the FFC, FHC and parent C3H mice are respectively 67, 35 and 0 per cent. Testing the null-hypothesis that the radiosensitivity of the sub-passaged homoplasts is the same as in the FFC hosts (76 per cent at 4200 r, Table I), the probability of obtaining the results observed is shown in the last column of Table II. Except in the FFC hosts, this assumption is obviously invalid. Although small numbers of animals were used, the results indicate that no inherent change occurred in the tumour with respect to its radiosensitivity as a result of one passage through a relatively curable FFC host, and that the cure-rate reverted to that of the original C3H tumour in each genetic category.

TABLE II.—*Comparative Radiosensitivity in Various Hosts of the Tumour Following One Sub-passage in an FFC Hybrid.*

| Recipient host. | Dose *in situ*. | Number of mice. | Number cured. | Cures (per cent). | *p*.* |
|---|---|---|---|---|---|
| FFC | 4200 | 12 | 8 | 67 | 0·50 |
| FHC | 4200 | 14 | 5 | 35 | $<0 \cdot 01$ |
| C3H | 4200 | 10 | 0 | 0 | $<0 \cdot 001$ |

* *p* tests the hypothesis of " adaptive radiosensitisation " of the tumour (see text).

### DISCUSSION.

At least four distinct biological phenomena are now known to be associated with genetic and extrachromosomal factors operating in the host-tumour relationship. The susceptibility to tumour transplantation is a simple genetic dominant, shown by the uniformly successful transfer of the inbred parent strain tumour to all $F_1$ hybrids. However, once such a graft is established, its rate of growth is no longer contingent on host genetics *per se*, but is dependent on the maternal extrachromosomal influence, which, when present, exerts a significantly accelerating effect. Likewise, a maternal influence, presumably the milk agent, is sufficient to induce spontaneous mammary tumours in $F_1$ hybrid females, the incidence approaching that in the inbred parent strain, in spite of genetic dilution. It has here been shown that the radiosensitivity of a tumour transplant, in this case the C3H mammary adencarcinoma, is dependent on both genetic and maternal (extrachromosomal) factors in the hybrid host. It appears, however, that the two factors are not necessarily independent. In the case of the factor-free $F_1$ hybrid, a uniform and uncomplicated response is elicited which seems to be a clear expression of immunogenetic differences between host and tumour. The relative heterogeneity of response in the factor-harbouring $F_1$ is difficult to assess, but may possibly arise as a result of a variable interaction between the milk-factor and the genetically heterozygous constitution of the infant hybrid mouse.

Oughterson, Tennant and Lawrence, (1940) demonstrated the increased radio-sensitivity of the parent strain tumour in $F_2$ hybrids where overt resistance, due to genetic segregation, was apparent. However, it has now been shown that, even in $F_1$ hybrids, where such gross manifestations of immunity as spontaneous regression, or failure of subsequent tumour challenges to " take " in cured animals, are absent, the principle of increased radiosensitivity in the hybrid host still holds, and may be considered as evidence of subliminal host resistance to the graft.

Barrett and Deringer (1952) and Barrett, Deringer and Hansen (1953) have described an apparently permanent adaptive change, namely an enhancement of the tumour's ability to grow in resistant backcross mice, which occurs in the C3H mammary carcinoma as a result of one transmission through susceptible $F_1$ hybrid hosts. Using as a source of tumour a homoplast growing in a factor-free hybrid and known to be radio-sensitive, the hypothesis that the tumour was inherently radiosensitised, as a result of this sub-passage, was tested and found to be untenable. This does not, however, exclude the possibility that prolonged serial transmission through such hosts would eventually modify the stability and radiosensitivity of the tumour; but under the conditions of this experiment, the radiocurability of these homoplasts in the three genetic categories proved to be virtually identical with the response obtained with a directly passaged C3H carcinoma. It is concluded, therefore, that the reaction of the hybrid host to the parent strain tumour is the primary determining factor. A similar phenomenon, in which host-reaction seemed to determine the radiosensitivity of the tumour, was observed when induced radiosensitivity in previously attenuated homoplasts growing in C3H mice was lost when these were transplanted to other hosts (Cohen and Cohen, 1954).

### SUMMARY.

The median effective dose for the C3H adenocarcinoma growing in C3H hosts was shown to be 5700 r. The median effective dose for this tumour growing in factor-harbouring (C3H × CBA) $F_1$ hybrid hosts is 5100 r, and for the same tumour in the reciprocal (CBA × C3H) $F_1$ hybrids, presumably factor-free, is 3950 r. The differences between these groups are significant, although all three groups of mice were uniformly susceptible to the tumour and showed no overt manifestations of resistance. It appears, therefore, that the radiosensitivity of a tumour is a quantitative measure of subliminal host-resistance resulting from immunogenetic differences in the host-tumour relationship, including extra-chromosomal factors.

The mechanism involved was further elucidated when a tumour growing in a factor-free $F_1$ hybrid, and known to be curable with 4200 r, was sub-passaged to mice of the three genetic groups. The homoplasts responded to treatment in the same manner as the directly passaged parent strain tumour in the corresponding hosts, indicating that no inherent radiosensitisation of the tumour cells *per se* had occurred.

All the facilities required for the maintenance of the animals used in this investigation were generously provided at the South African Institute for Medical

Research by Dr. J. F. Murray, to whom we are deeply indebted. We are grateful to Mr. J. E. Kerrich, Department of Mathematics, University of the Witwatersrand, for invaluable advice on the statistical analysis of the data.

### REFERENCES.

Barrett, M. K., and Deringer, M. K.—(1952) *J. nat. Cancer Inst.*, **12**, 1011.
*Iidem* and Hansen, W. H.—(1953) *Ibid.*, **14**, 381.
*Idem* and Morgan, W. C.—(1949) *Ibid.*, **10**, 81.
Braestrup, C. B.—(1944) *Radiology*, **42**, 258.
Cohen, A., and Cohen, L.—(1953) *Brit. J. Cancer*, **7**, 231, 452.—(1954) *Ibid.*, **8**, 313.
Eichwald, E. J.—(1953) *J. nat. Cancer Inst.*, **14**, 705.
Finney, D. J.—(1952) ' Probit Analysis.' 2nd edition. Cambridge (University Press).
Oughterson, A. W., Tennant, R., and Lawrence, E. A.—(1940) *Yale J. Biol. Med.*, **12**, 419.
Peacock, A.—(1953) *Brit. J. Cancer*, **7**, 352.


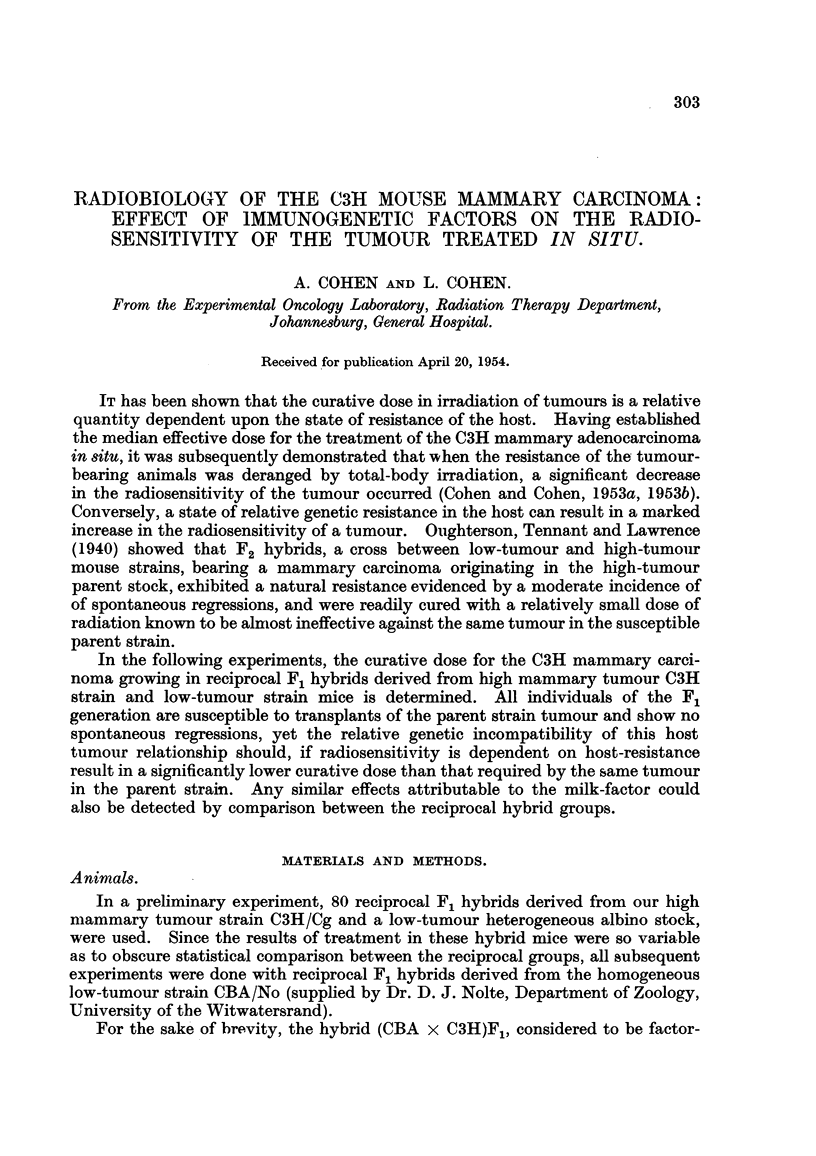

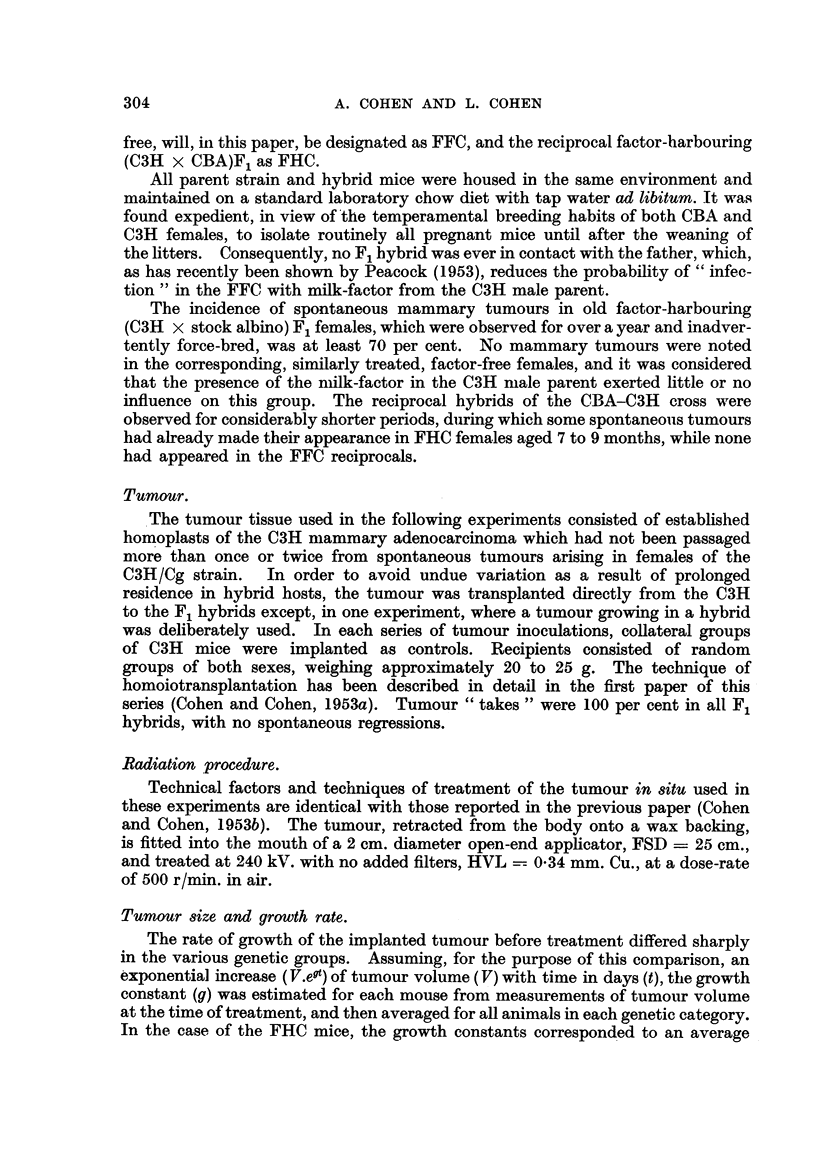

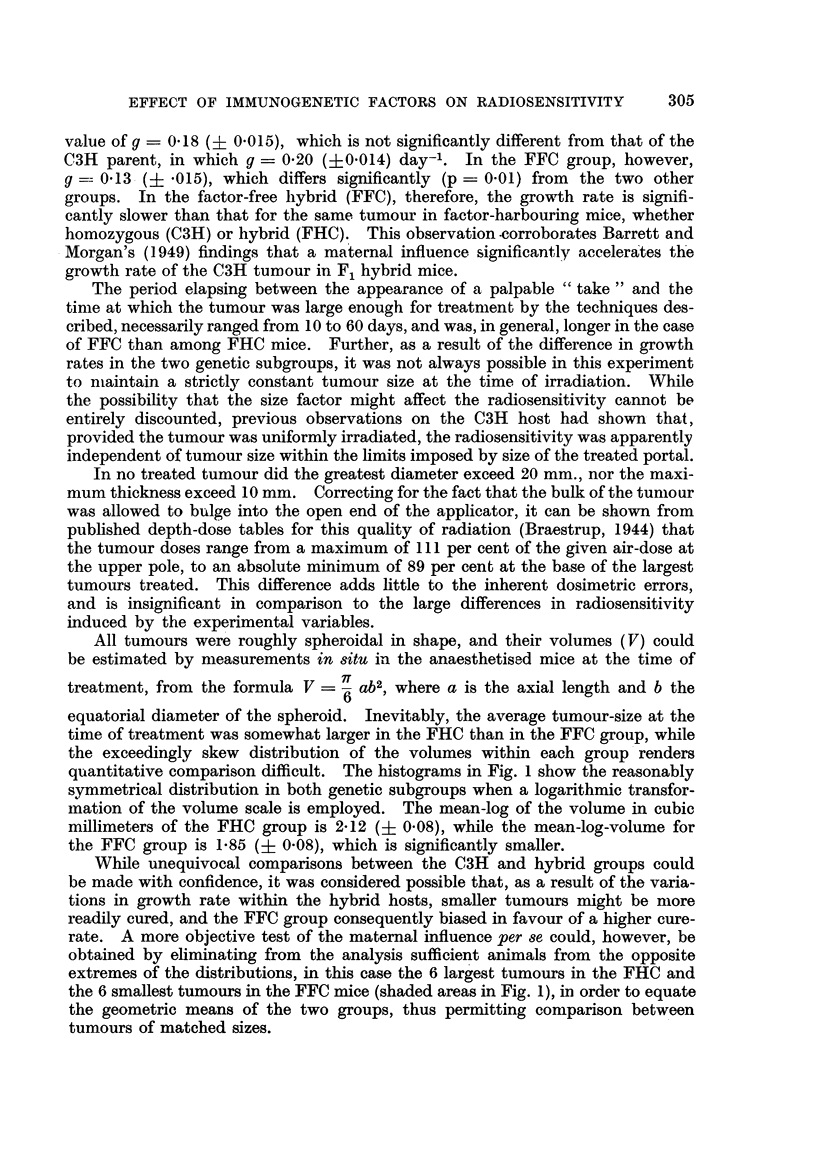

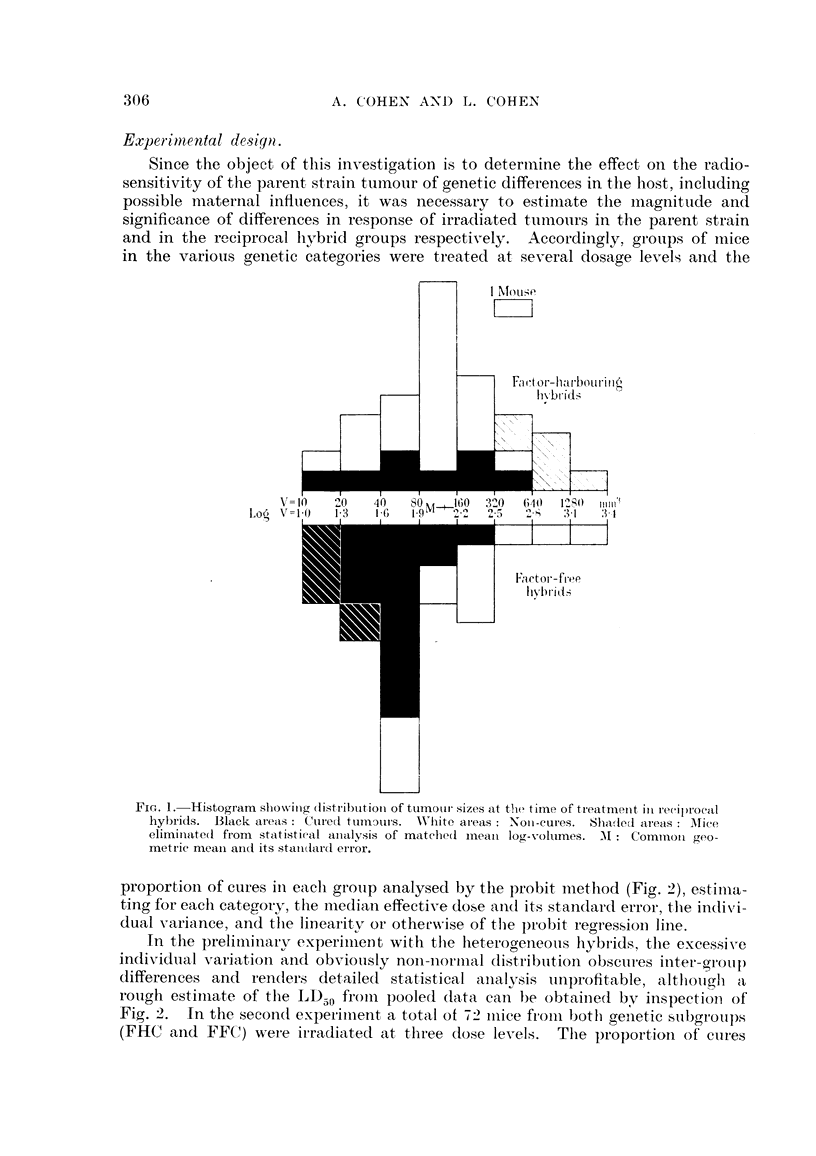

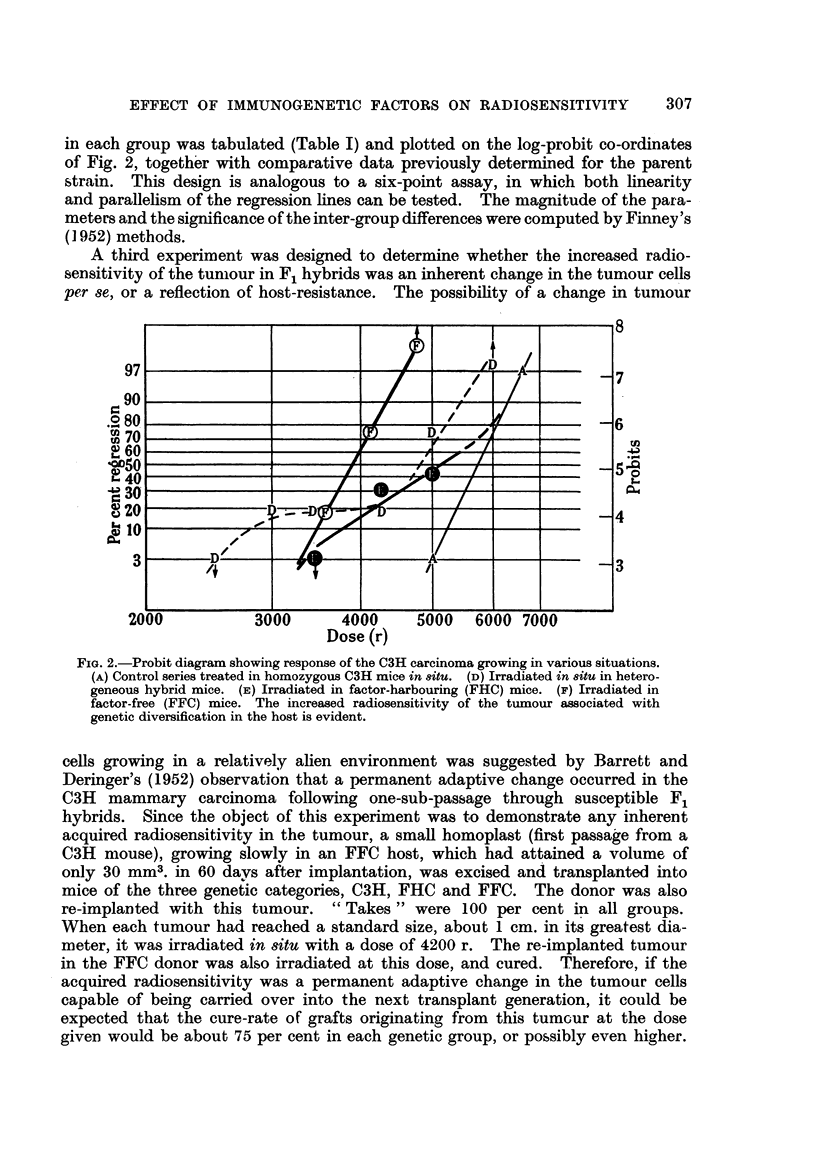

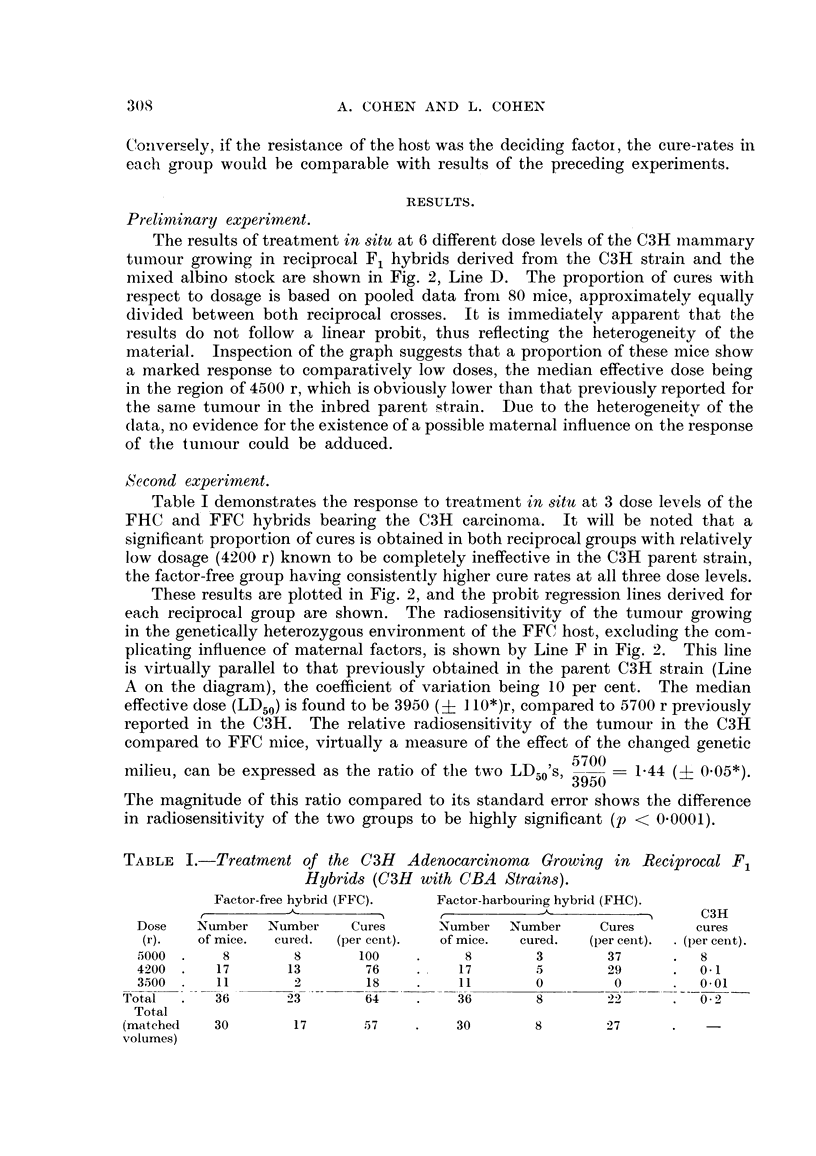

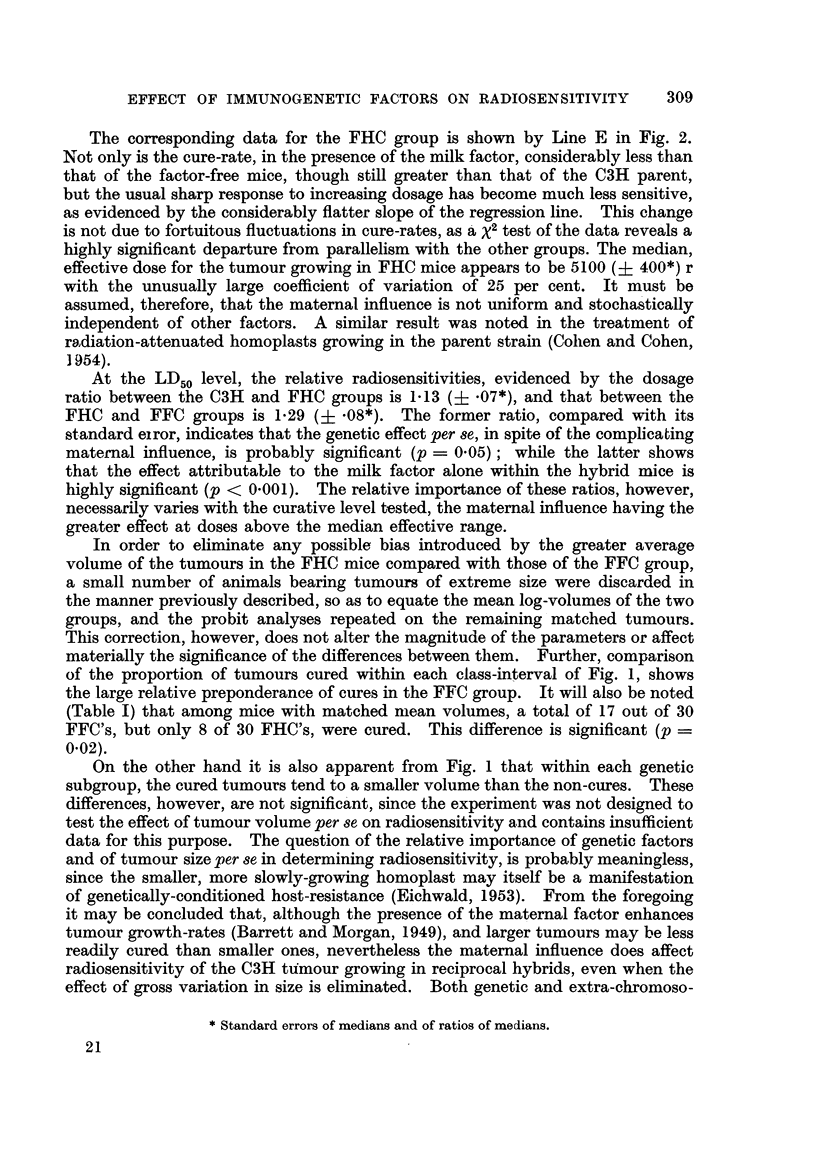

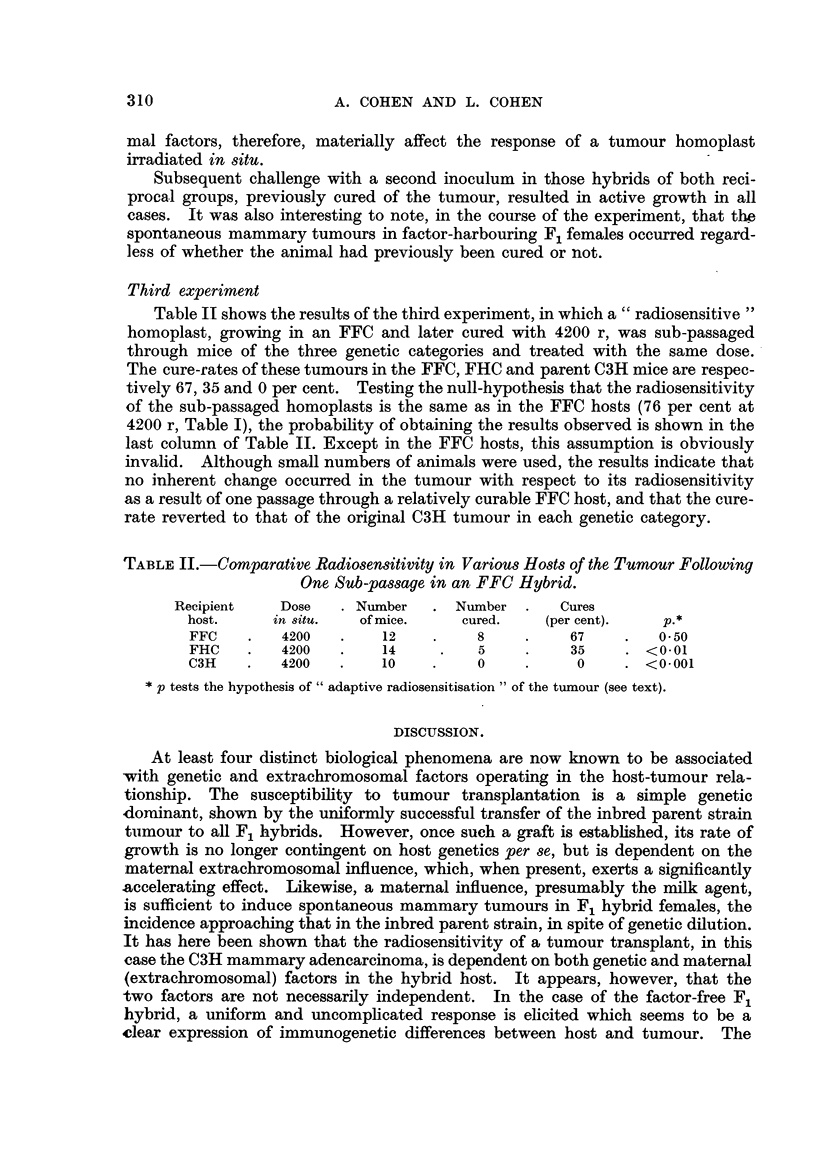

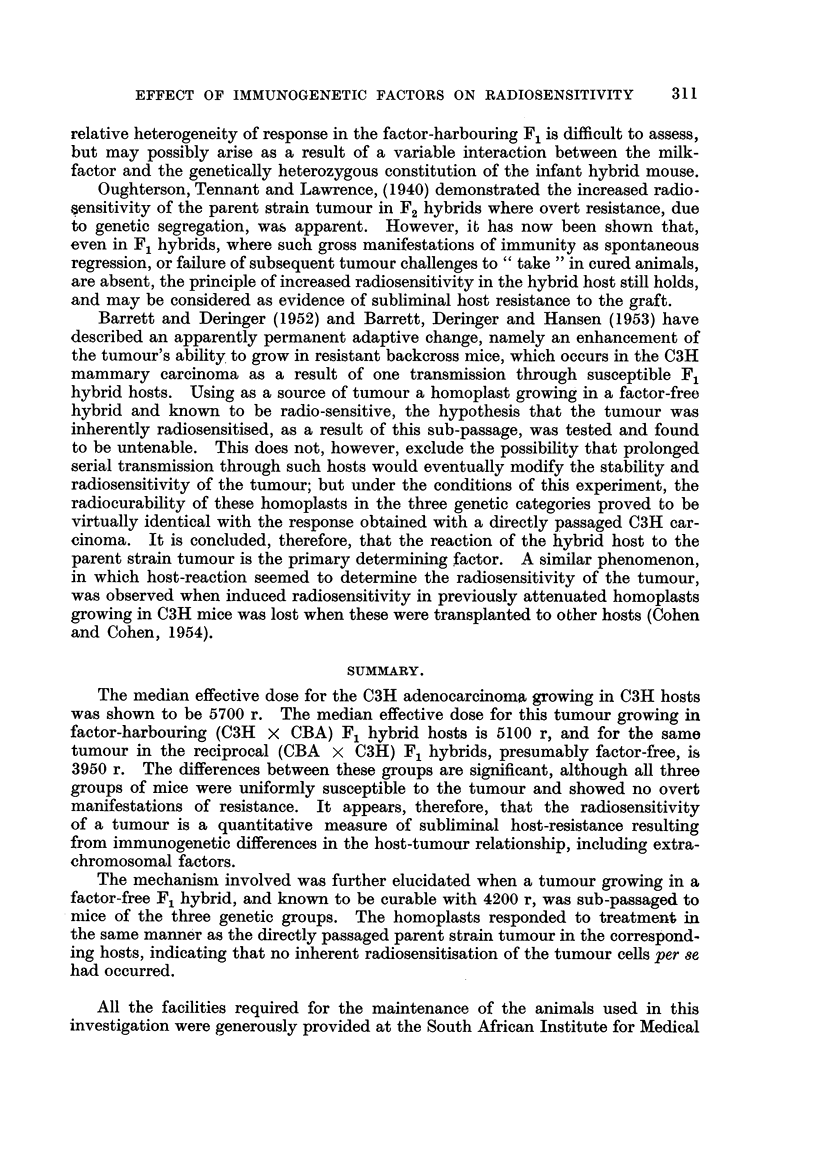

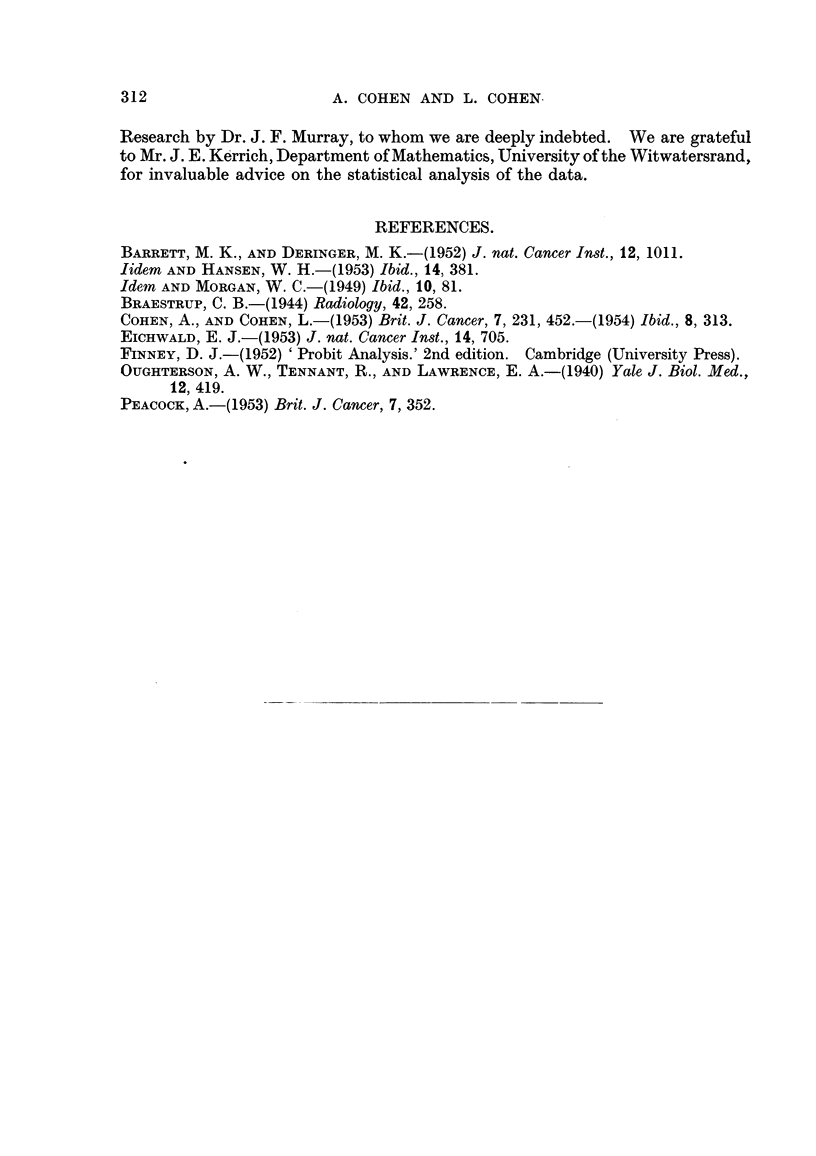


## References

[OCR_00680] BARRETT M. K., DERINGER M. K. (1952). Induced adaptation in a tumor: permanence of the change.. J Natl Cancer Inst.

[OCR_00686] EICHWALD E. J. (1953). Acquired immunity to the graft.. J Natl Cancer Inst.

[OCR_00694] PEACOCK A. (1953). A possible mode of transmission of the mouse mammary tumour agent by the male parent.. Br J Cancer.

